# The Cancer Parasite

**Published:** 1901-07

**Authors:** 


### THE CANCER PARASITE.

Dr. Gaylord, after a number of years of the most painstaking work in the cancer laboratory at Buffalo, reports that he has discovered the specific cause of cancer. He believes the organism to be indentical with the protozoon described by Plimmer of London, and by him demonstrated to be present in 1130 out of 1278 cases of cancer observed during a period of six years. The youngest forms of the parasite are not unlike *cocci;* older forms closely resemble fat droplets, but they do not respond to the ether and osmic acid tests. The fully matured organisms are spherical bodies with delicate outlines, in some respects similar to the ordinary connective tissue corpuscle. Bodies apparently identical with these can be demonstrated in the lesions of syphilis, pseudoleukemia and other diseases the cause of which are obscure. Dr. Gaylord explains that these diseases also are caused by protozoa, and that in time we shall, by the use of improved methods, be able to separate the various species, and assign each to its special disease.

Unfortunately, Dr. Gaylord has not been able to cultivate the organism outside of the body, and so far as we can learn he has not yet succeeded in reproducing typical carcinoma in animals by inoculation.

It is a remarkable coincidence that at about the time Dr. Gaylord's article was published, Schüller, of Berlin, in a short monograph, announced his discovery of an organism found to be constantly present in cancer, the life history of which he claims to have successfully studied. He has grown pure cultures outside of the body, and, by inoculation, has reproduced typical cancer in animals, and later recovered the germ from the lesions thus produced. He does not attempt to classify the organism, though he believes it is neither a yeast nor identical with Plimmer's body.

According to his report Schüller's parasite certainly seems to have satisfied Koch's postulates; but, as wisely suggested by a contemporary, we must bear in mind that Schüller is an interested witness, and therefore we should not accept his testimony on so important a matter until it has been confirmed by some other competent authority.

Whether the organism described by Schüller is identical with that of Plimmer and Gaylord, and, if so, whether it is really the cause of cancer cannot be definitely determined at the present writing. Much light, however, will be thrown upon the subject before many weeks have passed. Dr. Gaylord's second article is probably now almost ready for publication, and Schüller's sensational claims will no doubt be investigated within a short time. In the meanwhile, it seems wise to withhold judgment. W. B. R.

